# Autologous Iliac Crest Bone Grafts in Orbital Floor Reconstruction: A Quantifying Study of Graft Resorption and Clinical Outcome

**DOI:** 10.1007/s12663-025-02844-2

**Published:** 2026-01-27

**Authors:** E. M. Strabbing, S. A. J. Kronig, R. J. G. van der Mooren, M. Wevers, D. Paridaens, M. J. Koudstaal, E. W. Wolvius

**Affiliations:** 1 Department of Oral and Maxillofacial Surgery, Special Dental Care and Orthodontics, Erasmus University Medical Center, Dr. Molewaterplein 40, Rotterdam, 3015 GD The Netherlands; 2 Oculoplastic & Orbital Service, The Rotterdam Eye Hospital, Rotterdam, The Netherlands; 3 Orbital Service, Department of Ophthalmology, Erasmus Medical Center, Rotterdam, The Netherlands

**Keywords:** Orbital fractures, Bone grafting, Bone resorption, Bone remodeling, Iliac crest, Tomography

## Abstract

**Background:**

This study investigates bone graft resorption following orbital floor reconstruction with autologous iliac crest bone grafts. While autologous grafts are valued for their biocompatibility, concerns remain regarding unpredictable resorption and its potential impact on functional and aesthetic outcomes.

**Methods:**

Between 2009 and 2013, 32 patients with pure blowout fractures were treated with iliac crest grafts. Of these, 10 consecutive patients (median age 47.5 years) met inclusion criteria. CT scans were analyzed at two points: immediately postoperative (T1) and at 12 months (T2). Graft volumes, orbital volume, and clinical outcomes, including enophthalmos and diplopia, were assessed.

**Results:**

The mean initial graft volume of 903 mm³ decreased by 70% at T2. Despite significant resorption, orbital volume increased 3.2% due to bone remodeling. No clinically relevant enophthalmos or diplopia was observed. Donor site complications were minimal, with scarring being the most common.

**Conclusion:**

Significant bone graft resorption does not impair long-term outcomes in orbital reconstruction. Remodeling enhances stability, supporting autologous iliac crest grafts as a cost-effective option, particularly in resource-limited settings. Further research is needed to explore the clinical implications of graft resorption.

## Introduction

Orbital fractures are one of the most frequent types of facial fractures, following fractures of the nasal bone [1, 2]. The thinnest bone in the orbit is located at the medial wall and orbital floor, making the floor especially prone to fractures [2]. The decision for surgical repair depends on symptoms such as persistent diplopia, limited ocular movement, and clinically relevant enophthalmos (>2 mm), combined with CT findings of large defects or incarcerated tissue [2, 3]. The primary goal of surgery is to reposition the prolapsed periorbita and restore the orbital volume for functional and aesthetic outcomes [3].

Alloplastic materials, such as titanium mesh, are commonly used for orbital floor reconstruction due to their biocompatibility and contouring. However, they carry risks, including infection, rejection, and implant displacement. Malpositioned implants potentially lead to complications such as restricted extraocular motility, diplopia, and eyelid retraction [4]. Despite the growing use of alloplastic materials, authors still consider autologous bone grafts a viable choice for orbital floor repair due to their biocompatibility and osteoconductive properties [5–7].

One major disadvantage of autologous bone grafts is the need for a donor site associated with morbidity [8]. Additionally, bone grafts may be challenging to shape precisely to the orbital anatomy. Some studies suggest they can undergo unpredictable resorption, potentially leading to late complications such as enophthalmos [9]. While these concerns exist, only one study has specifically examined the extent of bone graft resorption in orbital wall reconstruction, finding partial resorption of iliac bone grafts without quantifying the resorption [7]. As a result, the impact of graft resorption on clinical outcomes remains uncertain. Therefore, the aim of this study is to evaluate the long-term outcomes of orbital floor reconstruction using autologous iliac crest bone grafts, with a particular focus on graft resorption. The specific objectives are: (1) to quantify the degree of bone graft resorption over a 12-month period using 3D imaging; (2) to assess changes in orbital volume resulting from bone graft resorption; and (3) to determine whether this resorption has any clinical impact on outcomes such as enophthalmos and diplopia.

## Materials and Methods

### Subjects

This study was performed in line with the principles of the Declaration of Helsinki. Ethical approval was obtained (C-2014358) for the present retrospective cohort study. This retrospective study included consecutive patients who underwent orbital floor reconstruction at the Erasmus University Medical Center, between February 2009 and December 2013. This specific timeframe was selected to ensure methodological consistency. During this period, a uniform surgical protocol was employed, specifically the routine use of monocortical iliac crest bone grafts for orbital floor reconstruction.

This consistency reduced the risk of confounding variables associated with evolving surgical techniques or materials introduced in subsequent years. Importantly, the timeframe precedes the adoption of newer 3D surgical technologies in the department, which have introduced some variability in procedures since then. Moreover, this period allowed for the collection of adequate follow-up data to assess long-term surgical outcomes using a single monocortical iliac bone graft.

Our treatment approach reflects a conservative policy. It is aimed at avoiding overtreatment while addressing functionally or cosmetically significant sequelae when they are present or clearly expected. In dislocated pure orbital fractures surgical repair was done in case of:

Persistent diplopia after the resolution of post-traumatic edema,
(Late) functionally or cosmetically disturbing enophthalmos, or.
Radiologically large fractures with significant prolapse of periorbita and clear orbital volume increase, where enophthalmos is anticipated and the patient prefers early surgical intervention rather than waiting for its clinical development.

The inclusion criteria were: (1) age > 18 years, (2) pure blowout fractures with an indication of orbital floor reconstruction using a single monocortical iliac bone graft, and (3) availability of both postoperative CT or cone beam CT (CBCT) scans, one within one-month postoperative (T1) and a second 12 months later (T2). Exclusion criteria included incomplete medical records, impure blowout fractures, trapdoor(-like) fractures and a history of prior orbital or eye muscle surgery.

In cases with medial wall fractures that lacked bony dislocation or soft tissue entrapment, reconstruction was limited to the orbital floor.

### Surgical Technique

Orbital floor access was achieved using a transconjunctival incision. After blunt dissection, the herniated orbital contents were carefully repositioned, and the bony defect was exposed and measured. The orbital floor defect was measured in the anteroposterior direction and in width using a ruler and orbital spatulas, respectively. A mark was placed on the spatulas to indicate the width, which was subsequently measured.

Monocortical autologous bone grafts were harvested from the anterior iliac crest using a 3–4 cm skin incision. A bone segment exceeding the maximal dimensions of the orbital defect, as determined clinically and on the CT or CBCT imaging, was harvested using osteotomes or an oscillating saw and subsequently contoured intraoperatively.

The graft was positioned on top of the defect with complete coverage of the defect margins, without the use of rigid fixation. Care was taken to ensure that the graft was placed posterior to the inferior orbital rim. In four cases, the bone graft was secured with a single Vicryl 3 − 0 suture passed through small drill holes in both the graft and infraorbital bony margin. Stability was achieved primarily through the anatomical fit and the compressive effect of surrounding orbital soft tissues. No intraoperative modifications or deviations from the standardized surgical protocol were required for any patient.

### Data Collection

Demographic information, trauma etiology, surgery indication, and time between trauma and surgery were collected.

### Bone Graft Measurements

CT data were analyzed by two investigators using the image post-processing software Mimics 17.0 (Materialise Inc, Ann Arbor, MI, USA). The raw DICOM datasets from CT or CBCT scans were imported into this software and examined in the 2D coronal, sagittal, and axial views. The CBCT scans made at T1 and T2 were obtained using the Iluma^®^ Cone Beam Computed Tomography Scanner (IMTEC Imaging, LLC). Coronal, sagittal, and axial views were evaluated during 3D segmentation to assess the full extent of the graft. Sagittal cuts were reviewed to confirm posterior extension and anatomical support of the orbital floor. Then, the skull was segmented using a predefined Hounsfield Unit threshold for the bone to create a mask of the facial skeleton. Region-growing techniques were used to separate disconnected bone structures, and a 3D model of the skull was generated for the first CT scan. The autologous bone graft in the initial postoperative scan (T1) was manually outlined and separated from the rest of the skull, with its volume automatically calculated and recorded.

**Fig. 1:**
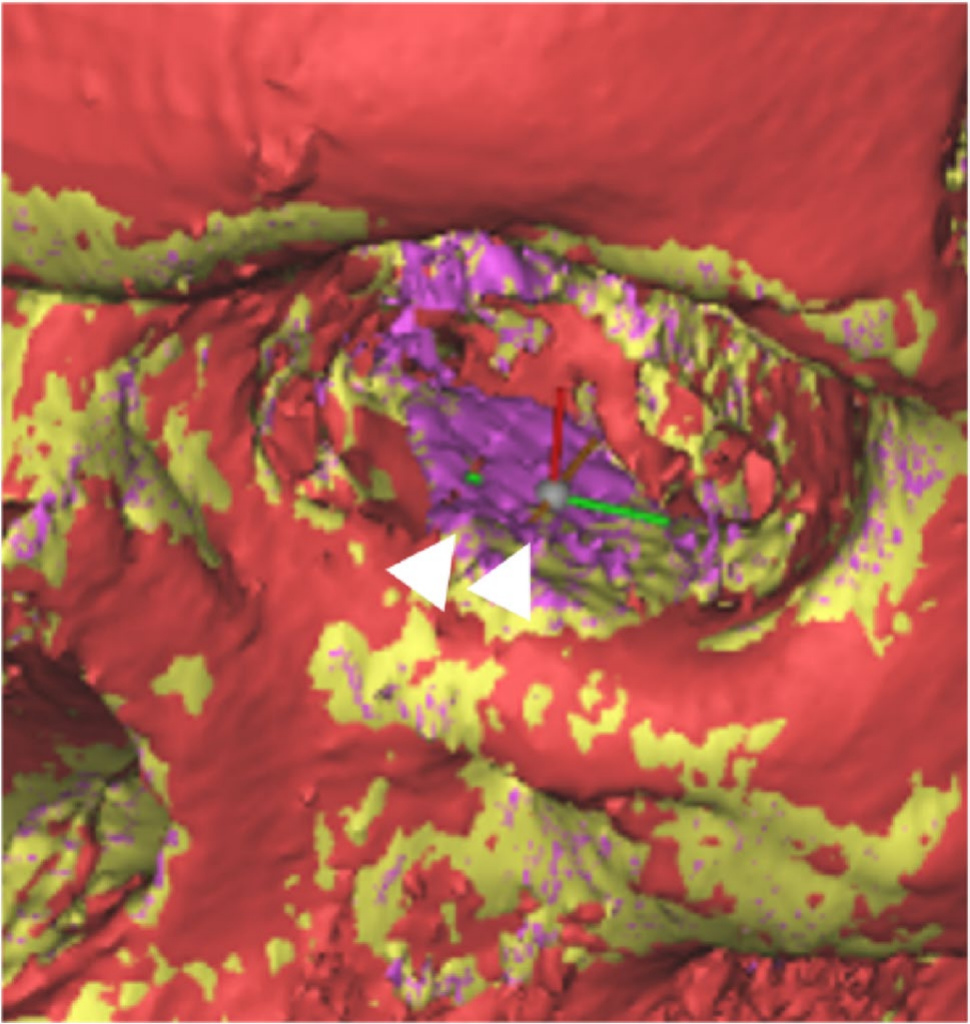
Analysis of bone graft resorption. 3D model immediately postoperative (T1, yellow) and 12 months postoperative (T2, red). A volumetric difference mask was generated to assess bone resorption between these two-time points. The arrows indicate the purple area, representing the volume difference due to graft resorption

The same procedures were repeated for the second postoperative CT scan. Automatic global registration was performed to superimpose the two 3D skull models. A mask of the volumetric difference between the two 3D models was then calculated (as shown in the purple region in Fig. 1). The autologous bone graft was outlined again, and its volume was automatically calculated.

### Orbital Volume

Orbital volume was calculated using Disior Bonelogic CMF Orbital software (Disior Ltd, Helsinki, Finland). The volume of the reconstructed orbits was measured immediately postoperative (T1) and after 12 months (T2).

### Clinical Outcomes

Clinical outcomes included the presence of enophthalmos (defined as >2 mm difference between eyes, measured with a Hertel exophthalmometer) [10] and diplopia (assessed using the Bahn and Gorman diplopia score) [11] at T2. Diplopia grades 3 and 4 were considered clinically significant [12]. The donor-side morbidity was also evaluated.

## Results

Between February 2009 and December 2013, thirty-two patients with a pure blowout fracture were treated with an autologous iliac crest bone graft. Ten patients met the inclusion criteria: eight males (80%) and two females (20%), with a mean age of 47 years (range: 27–73 years). Table 1 summarizes patient demographics, trauma aetiology, surgical indication, and time between trauma and surgery. 80% had isolated orbital floor fractures, and 20% had medial wall involvement without dislocation. The mean time between trauma and surgery was 11 weeks (SD 14 weeks; range 0–42 weeks). The first (CB)CT scan was performed at a mean of 8 days postoperative (SD 10 days), and the second (CB)CT at 12 months postoperative (SD 1 month). Two patients (patient 3 and 8) were classified as having large orbital floor defects, with estimated surface areas of approximately 2.5 cm² and 2.8 cm², respectively. These patients preferred early surgical intervention rather than waiting for clinical development of enophthalmos.

**Table 1:** Demographics of the patients

Patient	Age at trauma (years)	Sex (F/M)	Etiology	Indication for surgery	Time from trauma to surgery (days)
1	47	F	Violence	Diplopia	10
2	41	M	Fall	Enopthalmos	293
3	38	M	Violence	Large defect, high risk of enophthalmos	0
4	57	M	Work related	Diplopia	66
5	32	M	Work related	Enophthalmos, diplopia	27
6	48	M	Fall	Enophthalmos, diplopia	57
7	49	M	Violence	Hypoglobus, diplopia	13
8	27	M	Fall	Large defect, high risk of enophthalmos	5
9	59	F	Traffic accident	Enophthalmos, diplopia	220
10	73	M	Fall	Diplopia	68

### Rater Reliability

The interclass correlation coefficient was excellent at 0.99 (CI 0.91–1.00).

### Bone Graft Resorption

**Fig. 2:**
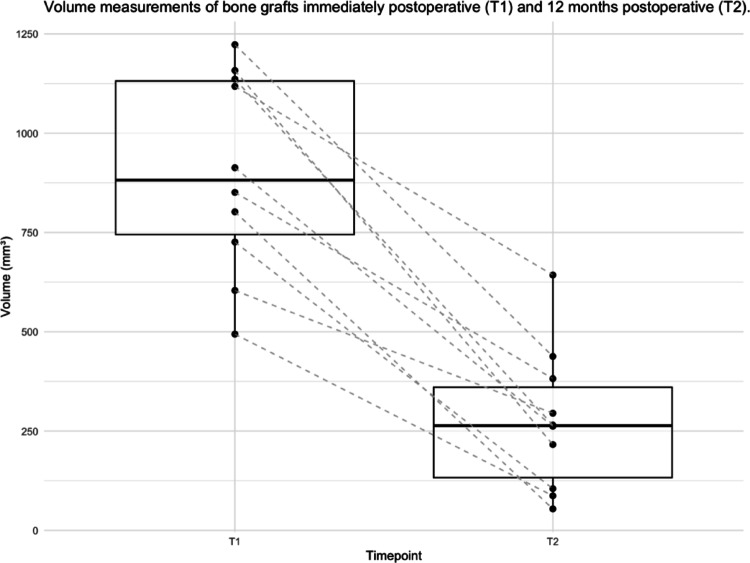
Volume measurements of bone grafts immediately postoperative (T1) and 12 months postoperative (T2). The box plot illustrates the volume measurements of bone grafts at two time points: immediately postoperative (T1) and 12 months postoperative (T2). Each black dot represents the volume measurement for a specific patient, with the dashed lines connecting the same patient’s data between T1 and T2 to indicate changes in volume over time. The box plot shows a reduction in bone graft volume from T1 to T2, indicating bone graft resorption over time

Figure 2 compares the initial postoperative bone graft volume (T1) and the final volume after 12 months of follow-up, measured in cubic millimetres (mm³) for each patient. The mean initial graft volume was 903 mm³ (SD 251 mm³), which was reduced by an average of 628 mm³ (SD 210 mm³), indicating a 70% reduction (SD 17%).

### Orbital Volume

**Fig. 3:**
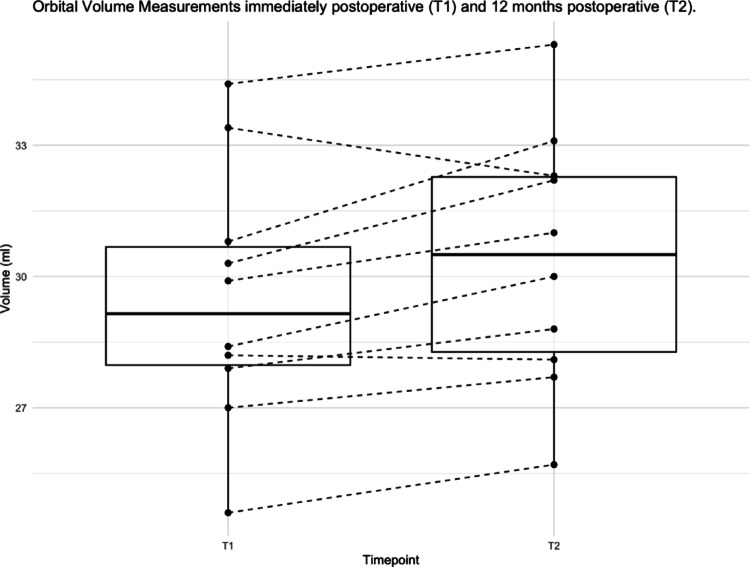
Orbital Volume Measurements immediately postoperative (T1) and 12 months postoperative (T2). Orbital Volume Measurements comparing immediately postoperative (T1) and 12 months postoperative (T2). The dashed lines connect each patient’s data point, illustrating changes in orbital volume over time. The box plots summarize the distribution of orbital volumes at both time points

**Fig. 4:**
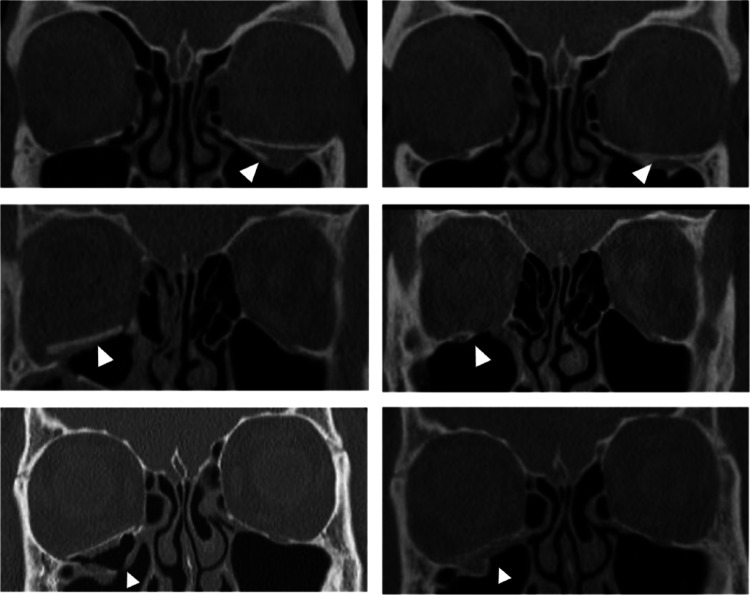
Changes in graft volume (immediately postoperative (T1) and 12 months postoperative (T2). The arrows indicate the location of the bone graft. First column: CBCT scans (coronal slides) illustrating the iliac crest bone graft in the immediate postoperative period (T1). Second column: corresponding CBCT scans (coronal slides) illustrating the iliac crest bone graft 12 months postoperative (T2). Bone remodeling and resorption are visible

Figure 3 presents the initial postoperative (T1) orbital volume measurements and final volumes after 12 months (T2). The mean initial volume was 29.5 ml (SD 2.9), which increased by 0.93 ml over the follow-up period. This represents an average orbital volume increase of 3.2%, primarily due to bone graft resorption, particularly at the graft edges. Figure 4 shows CBCT images of three patients, illustrating changes in bone graft volume over time.

### Clinical Outcome

No patients developed clinically relevant enophthalmos or diplopia 12 months postoperative (Table 2). Aside from a visible scar, no other donor site complications were observed at T2.

**Table 2:** Clinical outcomes 10–14 months postoperative (T2)

Patient	Hertel difference (T2, mm)	Diplopia score * (T2, 0–4)
1	1	0
2	2	0
3	0	0
4	0,5	0
5	1	0
6	0	0
7	0	0
8	1	0
9	1,5	0
10	2	1

*The Bahn and Gorman grading system was used to evaluate diplopia. The system categorizes diplopia based on its frequency of occurrence and whether it can be corrected, providing a clear framework for assessing the severity of the condition. The grading is as follows: Grade 1: Intermittent diplopia, only present when the patient is fatigued; Grade 2: Inconstant diplopia, occurring only during lateral or upward gaze; Grade 3: Constant diplopia, present during straight and level gaze but correctable with prisms; and Grade 4: Constant diplopia, present during straight and level gaze but not correctable with prisms

## Discussion

This study aimed to quantify bone graft resorption following orbital floor reconstruction using autologous iliac crest bone grafts and evaluate how this resorption affects orbital volume and clinical outcomes. We retrospectively analyzed a cohort of consecutive patients who underwent orbital floor reconstruction between February 2009 and December 2013. This timeframe was chosen because, during this period, a consistent approach to surgical techniques and materials was employed within our department, ensuring uniformity in the use of monocortical iliac bone grafts. Additionally, this dataset predates the department’s transition to newer 3D technologies and materials, which led to a shift in surgical practices. As such, it provides a unique opportunity to evaluate the outcomes of a widely used, cost-effective approach before these advancements.

Orbital floor reconstruction is generally indicated in cases of persistent diplopia, enophthalmos greater than 2 mm, restricted ocular motility, or radiographic evidence of significant orbital tissue herniation. Contraindications may include minimal fractures without clinical symptoms, poor general health status precluding surgery, or in cases where soft tissue prolapse spontaneously resolves without functional impairment [13]. Reconstruction can be performed using autologous bone grafts or alloplastic materials. Autologous bone, such as iliac crest, calvaria, or rib grafts, offers excellent biocompatibility and potential for integration but carries the risk of donor-site morbidity and unpredictable resorption. Alloplastic materials, including titanium mesh, porous polyethylene, and resorbable plates, eliminate donor-site complications and are easier to contour, but they can introduce risks such as infection, implant migration, and foreign body reaction [14].

While newer techniques and materials are now available, they come with significantly higher costs, which can limit their widespread implementation, particularly in healthcare systems with insufficient financial support. Therefore, the findings of this study remain relevant, as the approach studied offers a cost-effective option for settings with limited healthcare resources.

Our findings highlight a substantial bone resorption of 70% in autologous iliac crest bone grafts. However, despite this considerable resorption, no clinically relevant cases of enophthalmos or diplopia cases were observed in the patients included in the study. Bone graft resorption may occur through a combination of mechanical stress, ischemia, and osteoclastic activity in response to graft necrosis or remodeling. Endochondral bone, such as iliac crest grafts, is more prone to resorption compared to membranous bone [15]. Despite this, the present study found that resorption did not correlate with poor clinical outcomes, suggesting that adaptive remodeling may effectively maintain orbital support after initial graft placement.

This study reflects the diverse nature of orbital trauma, with a gender distribution showing a predominance of male patients (80%), likely due to their greater exposure to physical injuries, as noted in previous research [1]. The patients’ ages range from 27 to 73, and healing capacity and outcomes can be influenced by age, with younger patients generally expected to have better bone regeneration potential than older patients. The potential effects of age-related factors, such as bone density and tissue healing, on graft resorption and postoperative outcomes warrant further investigation.

In our study, diplopia and enophthalmos were the most common indications for surgery, suggesting that these are critical clinical concerns in the management of orbital trauma, as previously reported [2]. Additionally, the time interval between trauma and surgical repair varied significantly, ranging from 0 to 42 weeks. This variation could influence outcomes, particularly regarding late stage enophthalmos or diplopia. Further investigation is needed to assess whether delayed surgery impacts bone graft resorption.

Bone resorption is a well-documented concern when using autologous grafts for orbital reconstruction [16]. However, the implications of resorption in orbital floor reconstruction have not been extensively studied [17, 18]. Our study confirmed an average graft resorption of 70%, with no cases of complete graft loss. Importantly, this resorption did not negatively affect clinical outcomes, as no patient developed significant enophthalmos or diplopia. Comparisons between iliac crest grafts and other autologous grafts, such as calvaria or rib grafts, highlight differences in resorption rates due to variations in bone origin and anatomy [19]. Membranous bones from the calvaria are believed to undergo less resorption than endochondral bones, though this has yet to be proven in orbital reconstruction. Rib grafts have demonstrated minimal resorption and favorable outcomes in orbital fractures [20].

Interestingly, despite concerns that graft resorption could destabilise orbital volumes and increase the risk of late stage enophthalmos or diplopia, our findings indicate that resorption may contribute to beneficial remodeling, as reported in earlier literature [7]. It is essential to note that even with adequate volume correction, late-stage enophthalmos may still occur due to changes in soft tissue [3]. However, none of the patients in our study presented with late stage enophthalmos or diplopia at 12 months of follow-up, consistent with other studies reporting favorable long-term outcomes with autologous bone grafts [5, 21]. In addition, no other potential complications associated with orbital reconstruction, including infraorbital nerve injury, hematoma (from retrobulbar bleeding), infection, or persistent diplopia [22], were observed in this cohort.

Donor site morbidity, such as pain, scarring, or gait disturbance, is also a concern with autologous bone grafts [23]. In this study, donor-site issues were limited to visible scarring, aligning with previous literature on iliac crest harvest sites [7, 23].

Despite concerns about inaccurate graft placement leading to increased orbital volume and subsequent enophthalmos, none of the patients in our study experienced clinically significant enophthalmos or diplopia 12 months postoperatively. This suggests that while bone graft placement can be imperfect, functional outcomes remain satisfactory. However, it is worth noting that this study focused solely on orbital floor reconstruction, where placement precision may be less critical than in two-wall reconstructions. Furthermore, bone grafts may offer greater resilience in cases of a second trauma [9, 16].

To our knowledge, no other studies have focused specifically on the relationship between graft resorption and long-term clinical outcomes in orbital fracture repair. While a previous study [7] confirmed graft resorption and remodeling, it did not include quantitative volumetric measurements or systematically correlate these findings with clinical outcomes. Our study adds novelty by providing standardized CT-based volumetric analysis at defined postoperative intervals and demonstrating that, despite graft resorption, orbital volume increased without clinically significant enophthalmos or diplopia. In addition, we emphasize the cost-effectiveness of iliac crest grafts in resource-limited settings, an aspect not previously addressed in the literature. Nonetheless, the relatively small sample size and retrospective design limit the generalizability of our findings, and heterogeneity in patient characteristics and timing of surgery may have introduced confounding factors. Future studies with larger, stratified cohorts are warranted to determine whether delayed surgery, patient age, or bone quality influence graft resorption and long-term outcomes.

## Conclusion

In conclusion, while significant bone graft resorption occurs in the first year following the reconstruction of orbital floor fractures using autologous iliac crest bone grafts, this resorption does not negatively impact long-term functional or aesthetic outcomes. On the contrary, the remodeling process may offer advantages, contributing to the stability of the reconstruction. Further prospective studies are needed to understand the implications of graft resorption better and clarify the role of autologous bone in orbital fracture repair.

## Data Availability

The datasets used and/or analyzed during the current study are available from the corresponding author on reasonable request.
